# Antioxidant Activities and Selenogene Transcription in the European Sea Bass (*Dicentrarchus labrax*) Liver Depend, in a Non-linear Manner, on the Se/Hg Molar Ratio of the Feeds

**DOI:** 10.1007/s12011-021-02835-7

**Published:** 2021-07-31

**Authors:** Marinelle Espino, Harkaitz Eguiraun, Oihane Diaz de Cerio, José Antonio Carrero, Nestor Etxebarria, Iciar Martinez

**Affiliations:** 1 Research Center for Experimental Marine Biology and Biotechnology - Plentziako Itsas Estazioa (PiE), University of the Basque Country (UPV/EHU), Areatza 47, 48620 Plentzia, Bizkaia Spain; 2 Present Address: Aquaculture Department, Southeast Asian Fisheries Development Center (SEAFDEC/AQD), 5021 Tigbauan, Iloilo Philippines; 3 Department of Graphic Design & Engineering Projects, Faculty of Engineering in Bilbao, University of the Basque Country UPV/EHU, 48013 Bilbao, Bizkaia Spain; 4 Department of Zoology and Animal Cell Biology, Faculty of Science and Technology, University of the Basque Country (UPV/EHU), 48940 Leioa, Bizkaia Spain; 5 Department of Analytical Chemistry, Faculty of Science and Technology, University of the Basque Country (UPV/EHU), 48940 Leioa, Bizkaia Spain; 6 Basque Foundation for Science, IKERBASQUE, 48009 Bilbao, Bizkaia Spain; 7 Faculty of Biosciences, Fisheries and Economics, Norwegian College of Fishery Science, University of Tromsø, 9019 Tromsø, Norway

**Keywords:** Mercury (Hg), Selenium (Se), Thioredoxin reductase, Glutathione peroxidase, Selenogene transcription, Seafood, Fish, *Dicentrarchus labrax*

## Abstract

Feeding 3.9 and 6.7 mg Hg/kg (Se/Hg molar ratios of 0.8 and 0.4, respectively) for 14 days negatively affected *Dicentrarchus labrax* growth and total DNTB- and thioredoxin-reductase (TrxR) activities and the transcription of four redox genes (*txn1*, *gpx1*, *txnrd3*, and *txnrd2*) in the liver, but a diet with 0.5 mg Hg/kg (Se/Hg molar ratio 6.6) slightly increased both reductase activities and the transcription of *txn1*, *gpx1*, and *txnrd2*. Feeding 6.7 mg Hg/kg for 53 days downregulated the genes of the thioredoxin system (*txn1*, *txnrd3*, and *txnrd2*) but upregulated *gpx1*, confirming the previously proposed complementarity among the antioxidant systems. Substitution of 20% of the feed by thawed white fish (hake) slightly counteracted the negative effects of Hg. The effects were not statistically significant and were dependent, in a non-linear manner, on the Se/Hg molar ratio of the feed but not on its Hg concentration. These results stress the need to consider the Se/Hg molar ratio of the feed/food when evaluating the toxicity of Hg.

**Supplementary Information:**

The online version contains supplementary material available at 10.1007/s12011-021-02835-7.

## Introduction

A major concern in seafood production and consumption is the bioaccumulation of mercury (Hg) in species placed high in the trophic chain such as tuna, swordfish, marine mammals, and sharks [1, 2]. Hg is a powerful neurotoxin shown to exert adverse effects on the cardiovascular system and neurological development, mostly in fetuses, infants, and young children [3]. Although it is largely ignored in many risk–benefit assessment studies and reports, the neurotoxicity of methylmercury (MeHg^+^) has been mainly attributed to the irreversible inhibition of selenoenzymes provoked by its high binding affinity to Se [4]. The Hg’s affinity for Se is about 10^6^ greater than its affinity for S [3, 5]. Thus, the MeHg-cysteinate formed in the acidic conditions in the stomach is absorbed in the intestine where it shifts from being bound to thiol groups to binding the Se atom in selenol groups (R–SeH) of the amino acid selenocysteine (Sec) [6]. This results in the formation of MeHg-Sec complexes, diminishing the amount of biologically available selenium for selenoprotein synthesis [7].

The toxicity of Hg seems to be inversely related to the concentration of Se and directly related to Se/Hg molar ratio, as demonstrated by numerous studies [3, 8, 9]. The organic Se present in ocean fish has been shown to be bioavailable and effective in counteracting MeHg^+^ toxicity as long as there is a molar excess of Se with respect to the Hg [1, 5]. Several studies have also shown that the Se molecular form has relevance in neutralizing Hg toxicity [9, 10] and that the Se forms present in ocean fish, in particular selenoneine [9, 11, 12], may be more efficient and safe than other Se forms which, furthermore, display a very narrow range between an optimal and a toxic dose. The work by Yamashita et al. [12] is particularly interesting and shows how selenoneine increases the rate of MeHg^+^ demethylation and its excretion through the organic cations/carnitine transporter-1 (OCTN1). The formation of stable and inert complexes between selenolate and MeHg^+^ that can be excreted more efficiently than MeHg^+^ has also been published [13]. Indeed, the consumption of some species of tuna fish that usually have high levels of Hg and high Se/Hg molar ratios has not been linked to cases of Hg intoxication, while pilot whale consumption, with Se/Hg molar ratios below 1, did provoke intoxications in a Faroe Island population [5]. All these studies indicate that the magnitude of the risk of exposure and the severity of Hg intoxication are not predictable by the level of Hg alone, and one must take into consideration the molar ratio between Se and Hg and the Se molecular form.

Several indices have been proposed to estimate the Hg toxicity of seafood: from the simple calculation of the molar ratios of Hg and Se used by Wada et al. [8] to the more sophisticated Se health benefit value indices (HBVSe) proposed by Ralston’s group [1, 2]. Studies on the mechanism of Hg toxicity have exposed its influence on the activity of the reductase enzymes and of selenogene transcription in the liver in mammals. Thus, Hg inhibits the activity of the thioredoxin enzymatic system, which is involved in critical functions such as cellular regulation of the redox status [14], and includes thioredoxin (Trx) and the selenoenzyme thioredoxin reductase (TrxR).

The Trx-TrxR system is present in all organisms, and it is ubiquitously expressed in every cell type (see [14, 15] and references therein). Oxidized Trx is reduced again by TrxR with NADPH acting as the electron donor [14]. TrxRs display a broad substrate specificity [15] and were initially considered to be the only enzymes able to reduce Trxs [14]. However, it was later shown in human carcinoma HeLa [16, 17] and neuroblastoma [18] cells that when the TrxR activity is lost, the glutaredoxin 2 (Grx2) system, playing a backup role, is able to reduce both cytosolic Trx1 and mitochondrial Trx2 using glutathione in the presence of NADPH, indicating the need to consider both systems when evaluating Hg toxicity.

There are three TrxR isoenzymes in mammals: the cytosolic TrxR1, the mitochondrial TrxR2, and the testis-specific TrxR3 [14, 15]. However, a study on thioredoxin systems by Pacitti et al. [15] has found only two TrxR forms present in fish, the mitochondrial TrxR2 and an ancestral TrxR3, from which the authors hypothesize that the vertebrate cytosolic TrxR1 and testis-specific TrxR3 likely evolved via a gene duplication event. Interestingly, the same work also showed the presence of two thioredoxin reductase 3 (*txnrd3*) and another two thioredoxin 1 (*txn1*) gene isoforms in rainbow trout whose transcription levels varied depending on the tissue and were modulated by exposure to Se compounds and to exposure to infectious agents [15].

As already mentioned above, another relevant enzyme system found in most living organisms involved in the detoxification of reactive oxygen species (ROS) is the glutathione peroxidase, which includes the selenoenzyme glutathione peroxidase 1 (Gpx1). GPxs, by definition, have as a reductant a glutathione molecule; however, molecular studies showed that the majority of them had a shift in specificity during evolution, from thioredoxin or related proteins to glutathione defined by a specific motive [19]. For those cases, it has been proposed to name them thioredoxin GPx-like peroxidases (TGPx) [19]. In general, Gpxs are a target for Hg toxicity in mammals [20] and fish [21].

The expression of selenogenes is also affected by MeHg^+^. A unique feature of selenogene mRNA is the presence of a UGA stop codon within the open reading frame [6]. In conditions with sufficient Se available, the premature stop codons of selenogenes are translated to Sec [6]; however, with a deficiency of Se, the within-frame UGA codon is identified as a stop codon, and the mRNA proceeds to the nonsense-mediated decay (NMD) pathway [22]. Hg has been shown to decrease the expression of selenogenes in all the species examined, including zebrafish [6] and human hepatoma cells [23].

Given the scarcity of studies on the mechanism of action of MeHg^+^ ingestion on the selenogene expression of commercially relevant species, this research was undertaken with the main purpose of generating novel knowledge on such effect on European sea bass. Moreover, since the natural diet of carnivorous fish is other fish and the detoxifying effect of Se is dependent on the Se molecular forms (which may vary from one species to another) [12], we found interesting to examine the potentially mitigating effect of white fish ingestion on MeHg^+^ toxicity on European sea bass. As mentioned above, the positive effect of pelagic fish on MeHg^+^ toxicity has been well-documented [9, 11, 12], but that is not the case for white fish, and although adult European sea bass prefer small pelagic fish [24], it would forage on any small fish, including small hake when along the coast. Similarly, as mentioned above, the middle- and long-terms effects of Hg compounds on reductase activities and selenogene transcription have been previously documented, but we were mostly interested in documenting the effect of a short-term contamination, i.e., the response of these systems during the early stages of an accidental contamination taking into account the Se/Hg molar ratio of their feed.

Accordingly, our hypotheses were (1) that the ingestion of MeHg^+^ affects relevant biomarkers (in particular related to fillet trace element composition, hepatic reductase activities, and selenogene transcription) in the European sea bass and (2) that the substitution of 20% of the feed with a white fish (hake) would mitigate the effects provoked by MeHg^+^.

## Materials and Methods

### Ethical Aspects.

The experimental design was carried out in accordance with the EU Directive (2010/63/EU) for animal experiments. It was performed on European sea bass (*Dicentrarchus labrax*) and had been approved by the Ethical Committee for Animal Welfare (No. CEEA/039/2015).

The lowest number of fish that would provide meaningful results was selected according to our previous research [25]. We used 7 fish/tank for an initial 14-day experiment (Phase A) and 17 fish/tank for a subsequent 53-day experiment (Phase B) to allow for possible mortality during the longer experimental period. To minimize fish manipulation and the consequent stress, the fish lengths were not measured at the beginning of the experiments nor were the fish individually labeled. As a consequence, the average Fulton’s condition factor (CF = 100 W/L^3^, where W and L and fish weigh (g) and length (cm), respectively) of the fish could only be calculated at the end of the experiments. The specific growth rate (SGR) of the groups was estimated according to the following equation: SGR = 100[(log final body weight − log initial body weight)/days].

### Experimental Setup and Spiking of the Feed

The experimental setup and spiking of the feeds with MeHg^+^ are described in Eguiraun et al. [26]. A 14-day preliminary experiment (Phase A) was conducted to test the effect of three pre-selected MeHg^+^ doses and to identify the highest tolerable dose to be used in the subsequent Phase B. The exposure period of Phase B was 53 days, and it was intended to examine the potential mitigating effect of TH on the toxicity elicited by the intake of 6.7 mg Hg/kg feed on: (i) fish growth, (ii) Se/Hg molar ratio, (iii) hepatic reductase activities, and (iv) hepatic transcription of the selected selenogenes.

Prior to the beginning of the experiments, the fish were acclimated to the tanks for 3 days. The tanks (100 cm × 100 cm × 90 cm) were filled up to 80.5 cm of height with 810 L of aerated circulating seawater. Each tank was under direct artificial light (2 × 58 W and 5200 lm) to avoid shadows, with a 12 h/12 h dark/light photoperiod. The fish were fed once a day following the manufacturer’s specifications for their size, weight, and water temperature. No abnormal behaviors were observed during the experimental periods.

For Phase A, four experimental cases consisting of 7 fish/tank were used (Table 1). The control group (A1) was fed standard INICIO Plus from BioMar (56% crude protein, 18% crude fat), and the experimental groups A2, A3, and A4 were fed increasing doses of MeHg^+^-contaminated feed. The feed was spiked with MeHgCl, intended to provide 0.4, 4, and 8 mg Hg/kg feed for groups A2, A3, and A4, respectively. These values are within the range previously used by other authors [27] and lower than some occasionally encountered in heavily contaminated fish and whales [1].

**Table 1:** Weight (g) of the fish at the beginning and at the end of the experiment, which was 14 days for groups A1–A4 and 53 days for B1–B3. Statistical differences were calculated using the Student’s *t*-test. Se/Hg, Se/Hg molar ratio in the feeds; %IW, % increased weight of the group; CF calculated Fulton’s condition factor (CF = (W/L^3^) × 100) for the fish at the end of the experimental phases. SGR, estimated specific growth gate (SGR = 100[(log final body weight − log initial body weight)/days]

		***n* fish/**	**Weight (g) avg ± std**		**%**		
Group	**Se/Hg**	**tank**	**Initial**	**Final**	**IW**	**CF**	**SGR**
A1	29.5	7	141.6 ± 19.2	185.5 ± 40.4*^,a^	23.51	1.28 ± 0.12	0.79 ± 0.31^a^
A2	6.6	7	138.6 ± 34.9	180.2 ± 10.8*^,a^	23.10	1.36 ± 0.08	0.89 ± 0.61^a,c^
A3	0.8	7	141.6 ± 18.2	144.2 ± 24.4^b^	1.85	1.26 ± 0.15	0.03 ± 0.34^b^
A4	0.4	7	144.0 ± 22.5	162.9 ± 32.1*^,a,b^	11.59	1.32 ± 0.08	0.40 ± 0.28^b,c^
B1	29.5	17	177 .1 ± 37.6	218.4 ± 49.4*	22.98	1.32 ± 0.07	0.17 ± 0.03
B2	0.4	17	180 .9 ± 30.1	226.0 ± 46.1*	24.88	1.33 ± 0.11	0.18 ± 0.05
B3	0.4	17	174 .7 ± 35.4	214.4 ± 39.3*	20.32	1.30 ± 0.07	0.15 ± 0.04

^*^The fish were significantly (*p* ≤ 0.05) heavier at the end than at the beginning of the experiments

^a,b,c^Different superscripts in the column indicate significant differences

The contaminated feeds were prepared by spiking commercial feed with ethanolic solutions of methylmercury(II) chloride (CH_3_HgCl; Sigma-Aldrich), containing 0.0125, 0.125, and 0.25 mg MeHgCl /mL. The three batches of contaminated feed were prepared inside a hood by carefully mixing to ensure an even blending of the commercial feed with the corresponding ethanolic solution in trays that were covered with dark plastic and let to absorb the contaminant during 3 days. Twice a day during this period, each tray was uncovered and its contents mixed and covered again. At the end of the third day, the pellets in the trays appeared dry, and we considered that the MeHg^+^ had been absorbed. This produced three batches of feed spiked to contain 0.4, 4, and 8 mg Hg/kg feed. One sample of each batch was kept for inductively coupled plasma mass spectrometry (ICP-MS) analysis in order to calculate the actual Hg dose used in the experiment and the contents of Se and other trace elements of the feed.

For Phase B, the highest dose (8 mg Hg/kg feed) was selected (Table 1). The feeds for the control group B1 were prepared by mixing, on a dry weight basis, 80% of commercial feed and 20% thawed hake (*Merluccius capensis* and *M. paradoxus*) fillets (TH). The treated groups received 80% spiked feed (to a nominal concentration of 10 mg Hg/kg) and 20% TH (B2) or commercial feed (B3).

After the exposure periods in both Phases A and B, the sea bass were sacrificed by an excess of anesthetic. The weight and length of the fish were measured, and the livers were removed, frozen in liquid N_2_, and stored at − 80 °C until analyzed for reductase activities and selenogene expression. In addition, five whole fish from each group of Phase B (B1, B2, and B3) were frozen whole and stored at − 45 °C for subsequent ICP-MS analysis.

### Hg and Se Analysis

The fish from groups B1, B2, and B3 were partially thawed, and a sample of epaxial white muscle was excised. Since these samples were very small, the two smallest samples were pooled; thus, 3 determinations were made for each group of 5 fish: 2 determinations of 2 pooled samples each and one determination of the largest, single fish. The rest of the samples were analyzed in replicates: uncontaminated and contaminated feeds (*n* = 2 each) and TH (*n* = 4). All the samples were lyophilized (0.01 mbar, − 80 °C, 48 h) in a Cryodos-50 (Telstar S.A., Spain) freeze dryer and grounded with a commercial mill. The dried samples were stored in polypropylene tubes at 4 °C in the dark until analyzed.

Sample digestion and ICP-MS analysis were performed as described by Liñero et al. [28]. The metals were extracted by a microwave (Multiwave 3000, Anton Paar) acid digestion using a mixture of 2 mL HNO_3_ (69%, sub-boiling), 2 mL H_2_O_2_ (30%, tracepur; Sigma-Aldrich), and 2 mL Milli-Q. The clear solutions produced were filtered with 0.45 μm PVDF filters (Millex-HV, Millipore). Prior to ICP-MS analysis, appropriate dilutions of the extracts were gravimetrically carried out to 1% HNO_3_ in Milli-Q water. The extracts were stored at 4 °C in the dark until analyzed. Procedural analytical blanks were processed in a similar way in order to control the detection limit of the method.

ICP-MS (NexION 300X, Perkin Elmer) inside a class 100 clean room was used to measure ^202^Hg (in standard mode) and ^78^Se. The operating conditions were sample uptake flow, 0.4 mL/min; nebulizer gas flow, 0.90–1.00 L/min; plasma gas flow, 16 L/min; auxiliary gas flow, 1.2 L/min; RF power, 1600 W; dwell time, 50 ms; integration time, 1000 ms; 20 sweeps per replicate, and 3 replicates. Calibration standards were gravimetrically prepared with ± 0.00001 g of precision, starting from 1000 µg/L commercial solutions (Alfa Aesar, Specpure, Plasma standard solution) and acidified with sub-boiling 69% HNO_3_. The accuracy and precision of the method were assessed by means of the extraction and analysis together with the experimental samples of the DORM-4 (trace metals in fish protein) and SELM-1 (selenium enriched yeast) certified reference materials, both obtained from the National Research Council Canada (NRCC).

### Determination of Total DNTB-Reductase and Thioredoxin Reductase Activities

Hepatic total DNTB-reductase (representing the total reduction of DNTB in the presence of NADPH by TrxR, glutathione reductase, glutathione peroxidase, and other antioxidant enzymes) and thioredoxin reductase (TrxR) activities were measured in duplicate for each sample, with the Abcam TrxR Assay Kit (Abcam, product No. ab83463) as described below.

Frozen liver samples from 4 fish from each experimental group of both phases were partially thawed, and about 20 to 40 mg tissue was excised and homogenized in 200 μl of chilled assay buffer containing a protease inhibitor cocktail (Abcam, product No. ab65621) following the instructions of the manufacturer. The samples were homogenized in a Precellys homogenizer at 5,600 rpm, 2 × 30 s at 4 °C. The homogenates were then centrifuged at 10,000 g for 15 min at 4 °C. The supernatants were collected; aliquots were transferred to new tubes and stored at − 40 °C until analyzed. The total reductase and TrxR assays were carried out in 96-well microtest plate C (Sarstedt) in duplicates with 15 µL sample for the first replicate and 20 µL sample for the second. Two sets of assays were performed: (1) measurement of the total reductase activity (total DNTB reduction to TNB by the sample) and (2) measurement of DNTB reduction by TrxR in the sample with the use of the TrxR inhibitor included in the kit. The optical densities at 412 nm were read in Eon Microplate Spectrophotometer version 2.00.18.

The total protein in the supernatant was quantified by measuring the samples’ OD_280_ [29] using Epoch™ All-In-One Microplate Reader, and the protein concentration was estimated using the Protein OD_280_ application in BioTek’s Gen5™ Data Analysis Software. The activities were calculated as change in OD_412_/min/mg protein and plotted in the figures as percentage of the average activity of the control samples, A1 and B1 for Phase A and Phase B, respectively.

### Quantification of the Redox Gene Transcripts for Thioredoxin 1 (txn1), Glutathione Peroxidase 1 (gpx1), Thioredoxin Reductase 3 (txnrd3), and Thioredoxin Reductase 2 (txnrd2)

Total RNA was extracted from 60 to 80 mg of partially thawed liver samples (*n* = 4 from each experimental group) using the Invitrogen TRIzol™ Reagent following the instructions of the manufacturer. RNA yield, concentration, and purity were determined using Epoch™ All-In-One Microplate Reader and the nucleic acid quantification application in BioTek’s Gen5™ Data Analysis Software based on the OD_260_/OD_280_ ratio. Additionally, the RNA integrity was assessed with the Agilent 2100 Bioanalyzer and an Agilent RNA 6000 Nano Kit. The synthesis of complementary DNA (cDNA) was done using the Invitrogen SuperScript™ II Reverse Transcriptase (RT). All samples were synthetized at the same theoretical cDNA concentration, to a final concentration of 2 µg/µl.

The whole genome sequence of *D. labrax* is available at the National Center for Biotechnology Information (GCA_000689215.1, 30% of genome), with a new recently published version of it: GCA_905237075.1 (released 24th April 2021; 80% of coverage), but our targeted genes (*txn1*, *txnrd3*, *txnrd2*) were not identified in the genome at the time this work was performed. Only for *gpx* a EST was found DT044993. Therefore, to design suitable primers for the first 3 cases, homologous sequences of the target genes from closely related fish species (thioredoxin -XP_035530950.1; *txnrd1* (*txnrd1*)- XM_012823539-, *txnrd2*: -XM_012815943-; and *txnrd3* -Ensembl:ENSGMOG00000004823; XM_012829388; XM_007253309) were blasted (BlastN) against the nucleotide collection database limiting the search to sequences of *D. labrax* (tax ID: 13,489)*.* Resulting contigs were used for primer design. In the case of *gpx*, published DT044993.1 gene sequence was used. Primer3 (v. 0.4.0) software was used for primer design using default parameters and limiting the length of the amplicon to a maximum of 250 base pairs (Table 2). The primers were selected based on the following criteria: 18–24 bp in length, non-complementarity of the terminal nucleotide, with at least 50% GC content, and melting temperature (Tm) values between 56 and 62 °C. Further information on the identity of the sequences can be found in the Supplementary Material file. The sequence of the primers and the expected size of the amplicons are shown in Table 2. The primers were tested by conventional PCR using Taq polymerase (Invitrogen) chain reaction (PCR) in 50 µL reactions containing 1 × PCR buffer, 1.5 mM MgCl_2_, 0.2 mM dNTP, 0.8 µM forward primer, 0.8 µM reverse primer, 2.5 U Taq polymerase per reaction, and 2 µL of template cDNA in RNAse-free H_2_O.

**Table 2:** Primer design and SYBR Green qPCR parameters used to amplify target genes. The table indicates the ID of the sequences where the primers were designed; forward and reverse primer sequences (5′-3′sense); amplified amplicon size (bp); qPCR primer melting temperature (ºC); used primer concentration (µM); used cDNA dilution from the synthetized 2 µg in 20 µL; and the qPCR efficiency of each amplification reaction (%)

	**Primer design**		**qPCR parameters**				
**Gene**	**NCBI ID**	Primer sequence (5′-3′)	Amplicon (bp)	Tm (°C)	Primer conc (µM)	cDNA dilutions	E (%)
***txn1***	FN566754	Fw: CTGGTGGTGGTGGACTTCA Rv:CTCACATCCTCAGCCTCATCT					
			140	58	6.25	1/400	91.78
***gpx1***	DT044993.1	Fw: GCCCACCCTTTGTTTGTC Rv: GCACACTTTACTTGACCCTCTTG					
			238	55	6.25	1/400	86.75
***txnrd2***	GFJW01016941.1	Fw: TCTGTGTTATGGGCAGTTGG Rv: CCCGTCTCCTTGTTGAGTTG					
			247	55	3.125	1/5	132.78
***txnrd3***	GFJX01019781.1	Fw: CTCCGCAAATATGTCCCAGT Rv: GCACATTGGTCTGCTCTTCA					
			86	57	6.25	1/5	120.71

Amplifications were carried out in an Applied Biosystems 2720 Thermal Cycler with the following program: initial denaturing at 94 °C for 2 min followed by 35 cycles of denaturing at 94 °C, 30 s; annealing at 58 °C for *txn1*, 55 °C for *gpx1*, and *txnrd2* and 57 °C for *txnrd3* for 30 s; and elongation at 72 °C, 30 s. The program ended with a final extension step of 72 °C for 8 min, and the reactions were stored at 4 °C. The PCR products were analyzed by 2.5% agarose gel electrophoresis and their size estimated by comparison to a 50 bp DNA ladder (NZtech). The single amplicons obtained matched the expected size, and no dimmer products were certified in the non-template control sample added to the PCR. Additional confirmation of correct amplification was obtained by sequencing the amplicons in the General Genomics Service Sequencing and Genotyping Unit, of the SGIker service of the University of the Basque Country (UPV/EHU), and comparing them against the National Center for Biotechnology Information (NCBI) database. Amplicon similarities are available in Supplementary Table 1.

SYBR Green quantitative PCR (qPCR) was performed for gene transcription analysis in an Applied Biosystems 7300 Real-Time PCR System thermocycler (Applied Biosystems, Carlsbad, CA, USA). For normalization purposes, the method of Libus et al. [30] was used as described by Rojo-Bartolomé et al. [31]. Briefly, the cDNA obtained was quantified in the Synergy HT Multi-Made Microplate Reader (BioTek, Winoosky, USA) by Quant-iT™ OliGreen® stain (Invitrogen). The quantification was performed in a reaction volume of 100 μl with a theoretical cDNA concentration range of 0.02–0.2 ng/μL, at 485/20 nm excitation and 528/20 nm emission wavelengths. Real PCR input cDNA concentration was calculated using the high-range standard curve according to the manufacturer’s instructions.

The qRT-PCR was performed in separate MicroAmp® Optical 96-well reaction plates for each gene and cDNA sample. Non-template controls were run in each plate for quality control. The conditions for the qRT-PCR were first optimized regarding the cDNA and primer concentrations for each target gene (see Table 2). The amplifications were performed in 20 µL reaction volumes containing 1 × SYBR Green Master Mix (Roche, Basel, Switzerland) and primer concentrations each for forward and reverse primers of 6.25 µM for *txn1*, *gpx1*, and *txnrd3* and 3.125 µM for *txnrd2* and 2 µL of the selected cDNA dilutions (1:400 for *txn1* and *gpx1* and 1:5 for *txnrd3* and *txnrd2*). Each sample was analyzed in triplicate, and for each gene, a non-template control was added.

The cycling parameters of the qPCR consisted of an initial incubation at 50 °C for 2 min; followed by one step at 95 °C for 10 min; 40 cycles of 95 °C for 15 s and 55–58 °C for 1 min; and a final dissociation step at 95 °C for 15 s, 60 °C for 1 min, 95 °C for 15 s, and 60 °C for 15 s. After the RT-qPCR run, the standard curve, amplification plot, and melting curves were examined for the presence of outliers and of non-specific products such as primer–dimer artifacts. The CT values were then exported, and the mean, standard deviation, coefficient of variation, and the standard curve were computed. The RT-qPCR efficiency was determined by the formula E = 10^(− 1/slope)^. The CT values were normalized with the amount of cDNA loaded in the qPCR plates using for that a ΔCT formula adapted from the ΔΔCT normalization method described by Rojo-Bartolomé [31], and they were used to calculate the relative quantification of the transcription of the target genes.

### Data Analysis

The IBM SPSS Statistics software version 26 was used to calculate the average and standard deviation of the growth and SGR of the experimental groups, as well as statistical differences by the Student’s *t*-test between pairs of groups. Differences were considered significant when *p* < 0.05.

The same program was also used to calculate the average, median and quartile values, data spread, and distribution of both reductase activities and the fold changes in the transcription of the four redox genes. The data were tested for normality using Shapiro–Wilk, and the variances were checked for homogeneity. Kruskal–Wallis was used followed by pairwise comparison post hoc test to assess statistical differences in the reductase activities and gene transcription according to the MeHg^+^ treatment in both phases. Pairwise Pearson correlation coefficients and significance levels across all the variables studied were also calculated. Differences were considered significant when *p* < 0.05.

The data on the reductase activities were calculated as change in OD_412_ per min and per mg protein. They are plotted in the Results section as percentage of activity in the experimental groups with respect to their control groups, i.e., % activity in A2, A3, and A4 with respect to A1 and % activity in B2 and B3 with respect to B1. The red line in the box plots indicates the average normalized value (i.e., 100%) of the control groups (A1 and B1). Similarly, the representation and interpretation (upregulation or downregulation) of the fold changes values of the four genes were calculated as percentage of the difference of the median values of treated groups compared to their respective controls. The red line in the box plots indicates, as for the reductase activities, the average normalized value (i.e., fold change = 1) of the control groups (A1 and B1).

## Results and Discussion

### General Considerations

As already stated, the two phases of this study were not designed to be directly comparable. Phase A was intended to examine the short-term effect of increasing doses of MeHg^+^ on relevant liver parameters related to redox status and selenogene expression and to select the dose to be added to the feed in Phase B. Phase B was designed to examine the longer term effect of Hg on the same parameters and a potentially mitigating effect of raw white fish. Accordingly, the control group for Phase A was A1, and the control for Phase B was B1. The two phases will initially be discussed separately although the results have been plotted and tabulated together for convenience. It must also be remarked that due to the ubiquitousness of Hg, both the feed and the hake contained Hg, making it impossible to provide a real control group fed no Hg at all.

### Phase A

The calculated fish growth for each group, the Fulton’s condition factor at the end of the experiment, and the % increase weight during the 14 days are shown in Table 1. Mortality during the entire experiment was reduced to one fish that jumped out of the tank in A4.

Groups A1, A2, and A4 experienced a significant increase in growth during the 14 days the experiment lasted but not A3 which was significantly less heavy than A1 and A2 (Table 1). The extraordinarily low growth of A3 (1.85% of the initial weight) led us to further examine the biometric data of the four individual groups. For that, we estimated the specific growth rate of all individual fish assuming that the ranking of the fish according to their weight was conserved during the experiment. If the assumption was correct, all the fish increased in weight during the 14 days except for one individual in A2 and three in A3. The average SGR of A3 was significantly lower than those of A1 and A2 but not than the SGR of A4, which, in turn was only significantly lower than the SGR of A1 (Table 1). No significant differences were found among the CF of the 4 groups. It is however interesting to note that while the lowest CF was of A3 (as expected, due to the loss of weight of some of its individuals), the next lowest average CF corresponds to the control A1. Due to the distribution of weight of the fish in the two groups, we believe that the relatively low CF of A1 was due to the fact that the fish might have grown faster in length than in girth, while the low CF in A3 seems to be due to the fish not increasing in weight. Indeed, the lowest CF (1.04) in A1 was estimated to be displayed by the heaviest fish (220 g). The amount of Hg added to the feed was negatively correlated to the weight of the fish (Table 3) in agreement with the results of Stefansson et al. [32].

**Table 3:**
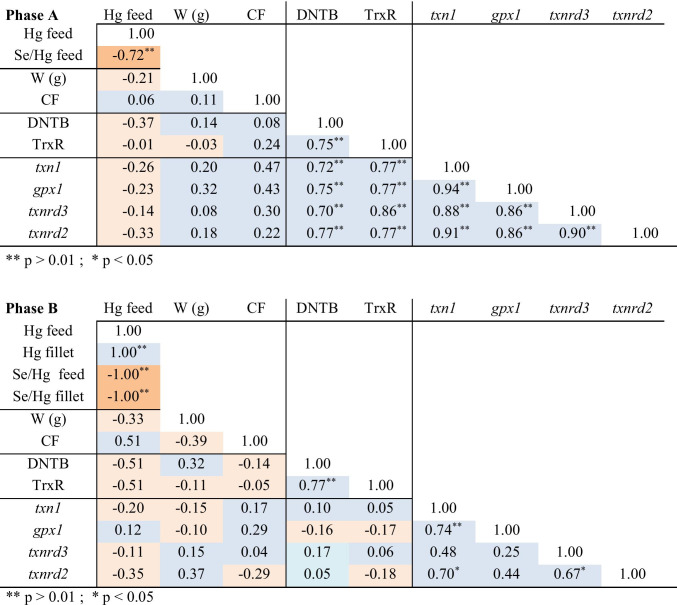
Pearson’s correlation coefficients among all the variables measured in Phase A and Phase B, as indicated. Hg feed and Hg fillet are the concentrations in mg Hg/kg feed and fillet, respectively; Se/Hg is the Se/Hg molar ratio; W is the weight of the fish (g); CF is the Foulton’s condition factor; DNTB is the total DNTB-reductase activity; TrxR is the TrxR activity; and *txn1*, *gpx*, *txnrd3*, and *txnrd2* are the transcription levels of the corresponding redox genes normalized with respect to the levels of A1 in Phase A and to the levels of B1 in Phase B. Cells in blue indicate positive correlations and in orange negative correlations. There were no significant differences among the parameters within each phase

We do not have an explanation for the lack of growth in A3. Although Hg content in the feed kept a negative correlation with the weight (Table 3), in this particular case, we do not consider the Hg to be the cause of the stunted growth in A3. The placing of the tank exerts a well-documented effect on fish growth [33], but we can disregard it in this study, given that the four tanks were placed together in a small room with restricted access to only one person, once a day, who was in charge of feeding them and recording their behavior. A disease outbreak is also discarded since all the fish came from the same stock and no abnormal features were observed during the post mortem dissection in any of the groups. Parasites were not observed either. Depressed growth may be the result of individual interactions taking place in a particular group that may lead to a general stressful situation for all of them. It is remarkable that the behavior of A3 was different from the behavior of the other three groups in Phase A, both during shoaling and in its schooling response as revealed by the analysis of their Shannon entropy published in a related work [26]. This result may indicate negative, stressful interactions among the individual fish resulting in hampered growth. It also confirms the convenience of developing non-invasive methods to monitor the behavior of the fish during farming, to serve as indicators of stressors or contaminants introduced in the system [34] and the suitability of the Shannon entropy of the fish system to provide that information. It is tempting to speculate that the deviated Shannon entropy of A3 reported in Eguiraun et al. study [26] and the abnormally low growth of the same group reported here may be the result of at least two stressors: MeHg^+^ toxicity and negative social interactions.

**Fig. 1:**
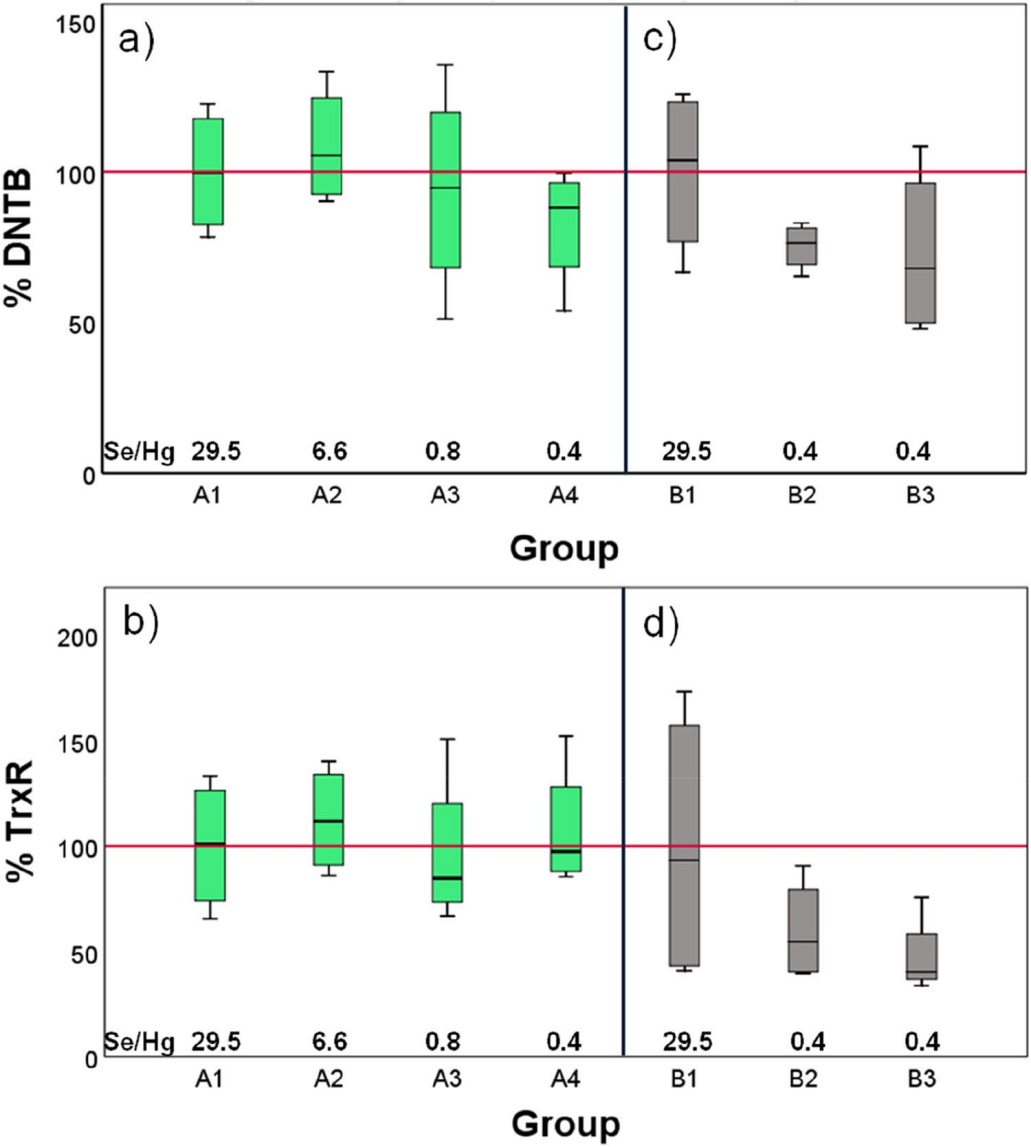
Boxplot of the effect of different Hg concentrations on the total DNTB-reductase (upper plot) and TrxR (lower plot) activities in the liver of *D. labrax* exposed for **a** and **b** 14 days (Phase A, in green) and **c** and **d** 53 days (Phase B, in gray). The groups received the following Se/Hg molar ratios in their feeds: A1 and B1, 29.5; A2, 6.6; A3, 0.82; and A4, B2, and B3, 0.4, as indicated in the graphs. Groups B1 and B2 received 20% TH in their diet. The results are plotted as percentage activity (as change in OD_412_/min/mg protein) of the median of the controls A1 and B1 for Phases A and B, respectively, *n* = 4. The red line represents the average normalized value of the control groups

In agreement with a previous study on *Diplodus cervinus* [21], both reductase activities were negatively correlated to the Hg content in their feed, and they were positively and significantly correlated to each other when considering all the experimental groups in Phase A (Table 3) or the groups with Se/Hg molar ratios below 1 (A3 and A4 in Fig. 1a, b). Interestingly, when the Se/Hg molar ratio of the feed was above 1, both activities were in fact slightly increased (A2 in Fig. 1a, b). No statistically significant differences were found in the total DNTB-reductase or TrxR activities among the four groups of Phase A. This lack of significance could be due to the short exposure time and the protective effect of Se present in the feed, which is similar to the findings of Branco et al. [35]. It could be hypothesized that there is a Se/Hg molar ratio threshold (which in our work would be somewhere between 6.56 and 0.82, corresponding to groups A2 and A3, respectively) (see Table 4) for the fish to maintain homeostasis: increasing concentrations of Hg until the threshold is reached would induce an increase in the activity of antioxidant enzymes, made possible by the molar excess of Se with respect to Hg. However, at Se/Hg molar ratios < 1, the fish would not be able to cope satisfactorily, and the activities would decrease, which may be the case in groups A3 and A4, which showed lower median values for both antioxidant activities although the differences were very small and displayed large inter-group variation (Fig. 1a, b). In agreement with these results, exposure to Hg has previously been shown to decrease the activity of the glutathione peroxidase [21].

**Table 4:** Results of the ICP-MS analyses and Se/Hg molar ratios in the samples. Average (Avg) ± standard deviation (std) values of the trace element concentrations in mg of element/kg dry matter

Sample	*n*	mg Hg/kg dry matter	mg Se/kg dry matter	Se/Hg molar ratio
Feed of A1	2	0.10 ± 0.01	1.17 ± 0.15	29.51 ± 3.09
Feed of A2	2	0.47 ± 0.03	1.22 ± 0.16	6.56 ± 0.49
Feed of A3	2	3.91 ± 0.22	1.26 ± 0.03	0.82 ± 0.02
Feed of A4	2	6.66 ± 1.06	1.08 ± 0.30	0.41 ± 0.05
B1, muscle	5	0.38 ± 0.01	1.04 ± 0.02	7.08 ± 0.36
B2, muscle	5	5.83 ± 1.32	0.87 ± 0.12	0.39 ± 0.11
B3, muscle	5	6.29 ± 0.54	0.91 ± 0.08	0.37 ± 0.04
Hake, muscle	4	0.27 ± 0.01	1.30 ± 0.10	12.17 ± 0.56

**Fig. 2:**
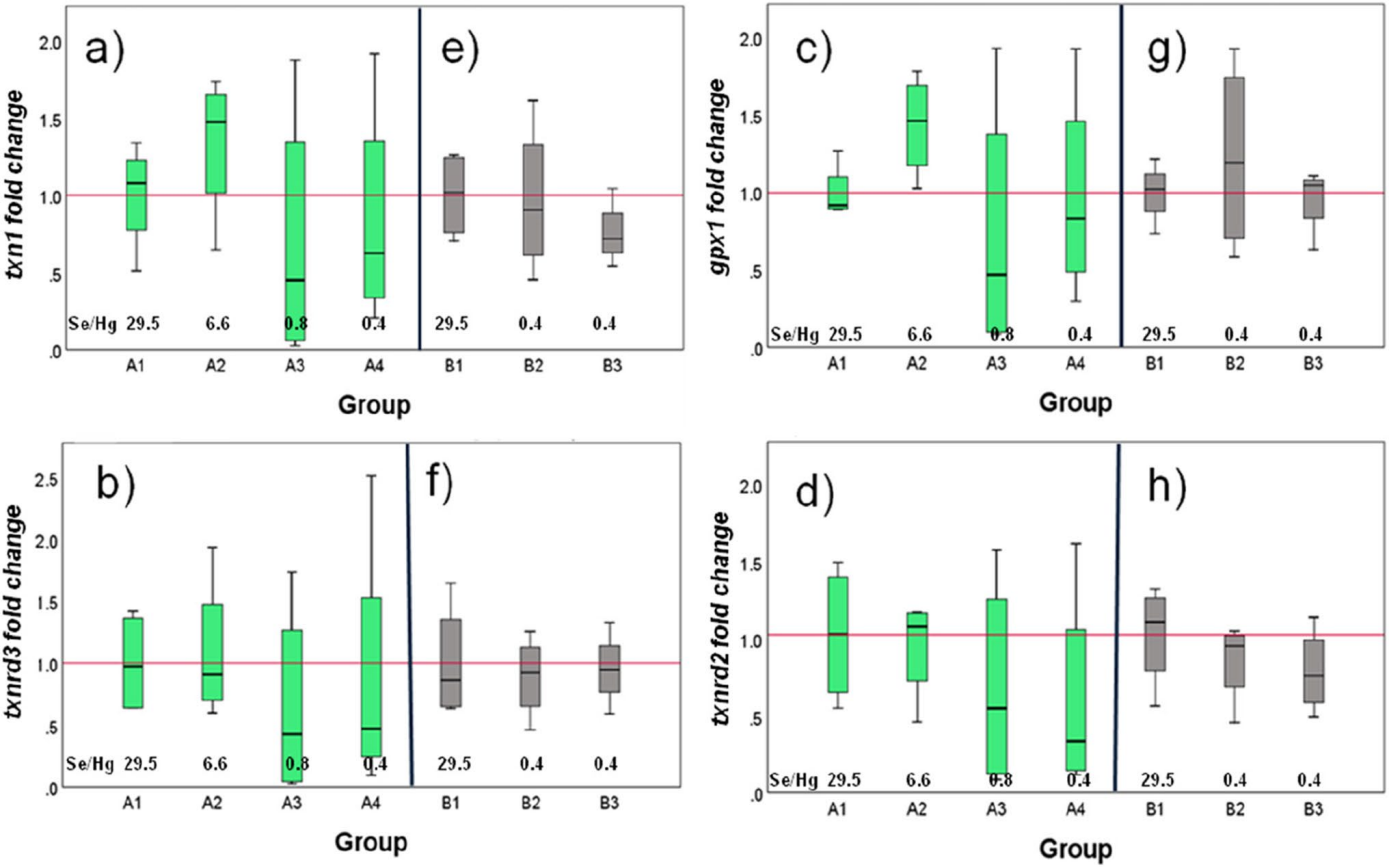
Boxplot of the effect of different Hg concentrations on the transcription levels of redox genes *txn1* (upper left); *gpx1* (upper right); *txnrd3* (lower left); and *txnrd2* (lower right) in the liver of *D. labrax*. Plots **a**, **b**, **c**, and **d** in green correspond to Phase A; plots **e**, **f**, **g**, and **h** in gray correspond to Phase B. The control for Phase A was A1, and the control for Phase B was B1. The groups received the following Se/Hg molar ratios in their feeds: A1 and B1, 29.5; A2, 6.6; A3, 0.82; and A4, B2, and B3, 0.4, as indicated in the graphs. Groups B1 and B2 received 20% TH in their diet. The results are plotted as fold change with respect to the median transcription values of the controls, *n* = 4. The red line represents the average normalized value of the control groups

The fold change in the transcription levels of the four redox genes *txn1*, *gpx1*, *txnrd3*, and *txnr2* in Phase A is shown in Fig. 2a, b, c, and d, respectively. As was the case for the reductase activities, the correlation between the transcription of the genes and the Hg in the feed was negative considering the four groups together (Table 3). However, the median values for the transcription of the hepatic selenogenes in A2, fed as A1 an excess of Se over Hg, were higher than that of A1 for *txn1* and *gpx1*, and it was only slightly different for *txnrd2* and *txnrd3* (Fig. 2). Under an excess of Hg, on the other hand, the transcriptions of all the genes were downregulated (Fig. 2).

The downregulation of genes upon exposure to Se/Hg molar ratios < 1 confirms the effect of Hg at the molecular level. These results agree with the works of Penglase et al. [6] in zebrafish embryos and of Branco et al. [23] in human hepatoma cells, who also found a downregulation in *gpx1* and *txnrd3* upon exposure to MeHg^+^. In a latter work [23], it was shown that exposure to MeHg^+^ slowed down the induction of antioxidant enzyme synthesis (which is also noticed in the present work) through the Nrf-2 pathway, a fundamental process in cellular response to states of stress, whose activation seems to be dependent on the Se/Hg molar ratios in the samples. In that regard, it is interesting to note that also the gene regulation response registered by our work seems to be dependent on the Se/Hg molar ratio of the feed and not on the Hg concentration alone. However, it must also be borne in mind that the response may be different depending on the stressor: cold temperature stress, for example, induced a downregulation of *gpx1* redox genes in the cyprinid *Tor tambroides* [36], but infection with a pathogen induced the expression at the transcriptional level of both *txn* and *txnrd* in rainbow trout [15].

The upregulation in the transcription of the genes *txn1*, *gpx1*, and *txnrd2* observed in A2 relative to A1 parallels a higher DNTB-reductase activity in these groups, while the increase in TrxR activity in A2 mirrors the upregulation of *txn1* and *txnrd2*. In Se-deficient groups (A3 and A4), the decrease in TrxR activity would reflect the downregulation of *txn1*, *txnrd3*, and *txnrd2*.

These results evidence that exposure to different concentrations of MeHg^+^ affect both the activity of reductases and the transcription of selenogenes. However, the lack of significant differences among the groups in terms of total DNTB-reductase and TrxR activities and transcription of the redox genes indicates that 14 days of exposure may be too short to observe significant toxic effects.

### Phase B

No mortality was registered in Phase B, and the three experimental groups, B1, B2, and B3, had significantly increased their weight by the end of the experiment (Table 1). Interestingly, the SGR and final CF were slightly higher in the group receiving both Hg and TH and lowest in the group receiving commercial feed and Hg, indicating a small but positive effect of the inclusion of raw fish in the diet of the sea bass on fish growth. It must be remarked again that the SGRs of Phases A and B are not directly comparable, because the growth rate of smaller individuals (Phase A) is usually different than that of larger fish and because the experimental setup and the lengths of time over which the SGR were calculated are different. The apparent lack of a statistically significant effect of MeHg^+^ intake on fish growth during Phase B further confirms the results of Stefansson et al. [32], who only noted a slight decrease in fish weight after 70 days of exposure.

The Hg and Se contents of the fish fillet were analyzed by ICP-MS. The accuracy of the method was assessed by the recoveries of each metal in the CRM and the precision by running three replicate CRM measurements on different days. The reference material, DORM-4 (tuna muscle), was analyzed together with the samples in each extraction series. Good recovery (80–111%) and RSD values (1–8%) were obtained. The recovery for the samples is expected to be comparable to that obtained with the CRM given the similarity of the matrix. Table 4 shows the average and standard deviation of the concentrations of Hg and Se (mg metal per kg dry matter of sample) for each group.

The discrepancy between the targeted (0, 0.4, 4, and 8 mg Hg/kg) and the actual (0.1, 0.5, 3.9, and 6.7 mg Hg/kg) concentrations of Hg in feeds was attributed to the fact that the commercial feed contained 0.1 mg Hg/kg (below the 0.2 mg Hg/kg legal limit for some feeds [37]) and to losses during the spiking of the feeds, as has been reported by other authors [32], and not to inaccurate measurements, since the recovery efficiency of the ICP-MS analysis for Hg was 97% (RSD 5% and *R*^2^ = 1.000). As expected, Hg concentration differed significantly (p < 0.05) between the spiked and unspiked samples, and the content of the sea bass muscle closely matched the concentration of Hg added.

The Se/Hg molar ratio of the samples is shown in Table 4. Feeds with 3.9 and 6.7 mgHg/kg and the muscle of sea bass fed with 6.7 mg Hg/kg had ratios below 1. The correlation between the content of Hg in feed and fillets was 1, as was the correlation between the Se/Hg ratio in feed and fillet, while the correlation between Hg content in either feed or fillet and Se/Hg in either sample was − 1 (Table 3). The excellent correlation between the Se and Hg concentrations in the feeds and in the fillet confirms previous works [38] and indicates that in 53 days the Hg was very efficiently bioaccumulated in the sea bass muscle. Table 4 shows that the Se content in the muscle also reflected that of the feed but only in the control B1 group. In the groups fed 6.7 mg Hg/kg feed, their concentration was lower, which might be explained if the fish was mobilizing Se from the muscle towards the liver and brain, which are more critical organs and common targets for Hg toxicity [35, 39]. It was noteworthy that in the muscle samples of the untreated European sea bass (B1) and of TH, the amounts of Se and Hg were inversely related (moles Se =  − 14 mol Hg + 35.01) although the relationship was not significant (*R*^2^ = 0.5887). This observation supports previous observations on the relationship between molar ratios of Se and Hg in carnivorous and omnivorous (such as sea bass) but not on herbivorous fish of the Madeira River, Brazil [40], as well as the potentially Hg-detoxifying effect of Se compounds documented by Yamashita et al. [12] in zebrafish embryos. Unfortunately, we could not analyze the Hg and Se content of the livers from the tested fish: they were small and the samples were used also for reductase and gene transcription analyses. However, based on published works [21, 41], we can reasonably assume that the concentrations of both of them will be higher in the liver than in the muscle.

No significant differences were registered in either the total DNTB-reductase or the TrxR activities between the control B1 and the two groups fed 6.7 mg Hg/kg feed (Fig. 1c, d). However, both reductase activities were negatively correlated to the Hg content (Table 3), and they suffered a non-significant decrease after 53 days of exposure to 6.7 mg Hg/kg. The inhibition of TrxR observed in the present work by 6.7 mg Hg/kg feed is in agreement with previous studies [21, 23, 35], indicating that the thioredoxin system is a toxicological target for MeHg^+^. Interestingly, the group fed 20% TH (B2) did show slightly higher reductase activities than the group fed only commercial pellets (Fig. 1c, d), indicating a protective effect of the nutrients from TH.

As in Phase A, the Hg content in the fillet was negatively correlated to the fish weight, to both reductase activities and to the transcription of three of the selected genes (*txn1*, *txnrd3*, and *txnrd2*), while the correlation to the transcription of *gpx1*, albeit small, was positive. Unlike in Phase A, where the transcription of the *txn1* and the 3 selenogenes was significantly and positively correlated to each other, in Phase B, there were only 3 significant positive correlations: between *txn1* and *gpx1*, between *txn1* and *txrnd2*, and between *txnrd3* and t*xnrd2*.

The transcription levels of *txn1*, *gpx1*, *txnrd3*, and *txnrd2* in Phase B are shown in Fig. 2 and in Table 5 and follow the same pattern as in Phase A for fish fed diets with a molar excess of Hg over Se: exposure to high Hg ratios downregulated *txn1*, *txnrd3*, and *txnrd2* and upregulated *gpx1*. With respect to B1, the downregulation in the transcriptions of B2 was in general less severe than in B3 (Table 5), indicating a protective effect of the inclusion of TH in the diet of these fish. The upregulation of *gpx1* in both groups may indicate complementarity among selenogenes from the antioxidant pathways. The glutathione system, particularly glutathione reductase, has been reported to complement reduced Trx and TrxR activities in the liver of zebra seabreams during Hg exposure [35]. Although that study did not examine the mRNA levels, our results support that complementarity at the transcriptional level by the negative correlation between the (lowered) activity of both reductases (Fig. 1c, d) and the (upregulated) transcription of *gpx1* (Fig. 2g).

The slightly different transcription levels of *txnrd3* and *txnrd2* may be a reflection of the different responses of the genes coding for the cytosolic and mitochondrial TrxR isoenzymes, respectively. Our results show that the mRNA levels of *txnrd3* were slightly increased upon Hg exposure compared to *txnrd2*, which is similar to the observations of Branco et al. [23]. This indicates a differential effect of MeHg^+^ on the transcription of the genes coding for TrxR isozymes which differ in location within the cell. The downregulation in the transcription of *tnx1* and *txnrd2* in B2 is parallel to the decrease in the total DNTB-reductase and TrxR activities of the group (Figs. 1and 2), indicating a net Se deficiency in these individuals.

In any case, there seems to be a clear trend in gene transcription between those groups with Se/Hg > 1 from those with Se/Hg < 1, in which the mRNA production is decreased. The main discrepancy is found in group B2 (Se/Hg < 1 and fed hake) which displays a large variation in response and whose median transcription value is higher than in the control. A possible explanation for this is that an increase in the transcripts of mRNA does not mean that the synthesis of corresponding protein is also increased: if there is not enough Se, the synthesis will stop at the UGA, and the Se-Cys will be channeled to the synthesis of more relevant selenoproteins [22]. This seems to be the case in our work, where the reductase activities are always lowered when the Se/Hg molar ratio is lower than 1.

Compiling both phases, our work, even though it is not directly comparable to that of Branco et al. [23] (since they used a mammalian cell culture and studied the effect of the addition of Na_2_SeO_3_, while our different Se/Hg ratios are caused solely by differences in the Hg levels of the feeds in the in vivo experiment), supports the model they proposed regarding the downregulation of the cytosolic and mitochondrial thioredoxin system (*txnrd3* and *txnrd2*) by exposure to MeHg^+^ and the relevance of the mitochondrial thioredoxin system (*txnrd2*) as target for Hg toxicity. Moreover, our results on the expression of *gpx1* in phase B appear to indicate a compensatory mechanism probably related to the expected increase in ROS.

To summarize, the effect of Hg administration seemed to be non-linearly dependent on the Se/Hg molar ratio of the feed. The administration during 14 days of feeds with a Se/Hg molar ratio < 1 slightly diminished the fish growth, the total DNTB-reductase and TrxR activities, and the mRNA levels of the four examined redox genes in the liver. A more prolonged 53 days of exposure to 0.4 Se/Hg molar ratio) induced an accumulation of Hg in fillet that rendered it unsuitable for consumption, and both reductase activities displayed lowered activities, but no significant decrease in growth was detected. The Se/Hg molar ratio of the feed was closely reflected in the muscle and seemed to modulate the response of the fish to Hg exposure: at Se/Hg molar ratios higher than 1, both reductase activities were slightly increased as were the mRNA levels of *txn1*, *gpx1*, and *txnrd2*, and only the transcription of *txnrd3* was slightly decreased. The present work shows an ability of the liver to cope with the intoxication for short periods of time, possibly by mobilizing Se from other, less critical tissues into the liver, or through the activation of complementary antioxidant pathways/systems shown by the slight upregulation of *gpx1*.

Our results support the hypothesis proposed by Branco et al. [42] indicating the usefulness of relevant components of the thioredoxin system as biomarkers of Hg toxicity, further indicating that the transcription levels of selenogenes alone may not be an appropriate measure of MeHg^+^ exposure and that the efficiency of the Se-dependent translation process may be the main determinant for the MeHg^+^ toxicity on the thioredoxin system. Moreover, our work indicates that the Se/Hg molar ratio must be taken into account to evaluate Hg toxicity. It was also interesting to note that the substitution of 20% of the feed with TH seemed to slightly counteract the negative effect of the ingested Hg. Given that the TH and the feed had similar levels of Se and keeping in mind the relevance of the Se forms to neutralize Hg toxicity (compare, for example, the narrow range of safety use when applying Na_2_SO_3_ [23] versus the higher and yet safe concentrations of Se in the blood of individuals who ingested selenoneine [9]), it would be highly relevant to map the Se forms present in the most common seafoods. It also remains to be investigated whether the changes and in particular the levels of Hg accumulated in the edible tissues might be reversible as shown in previous studies [35].

One relevant result of the present short-term experiment is that the classical parameters used to assess the effect of MeHg^+^ on selenoprotein transcription, expression, and activity did not reflect a significant deviation from the control, indicating the ability of the fish to cope with the toxicant under these conditions for the tested periods of time. This stresses the relevance of analyzing the fillet of all types of seafood (and not only that of harvested top predators) and to assess its Se/Hg ratio to ensure its wholesomeness, particularly in the light of the ubiquitous presence of Hg in the environment.

### Additional Considerations

The large variations noted in growth, enzymatic activities, and gene transcription within each treatment group can be attributed to several factors: one is the short time of exposure. As indicated in the Introduction, we were interested in documenting the effects of a short, potentially accidental exposure of fish to MeHg^+^, and although we were expecting the differences on the measured parameters to be significant under the conditions used, we found only trends, indicating the ability of the fish to cope under the conditions used in our work. Another factor is the low number of individuals analyzed, and further works in this field should indeed be performed using a larger number of individuals. However, in our experience, high individual variability is usually registered in undomesticated or recently domesticated species at different levels: from genetic diversity in *Pandalus borealis*, where 90% of the population genetic variability in the Barents Sea was registered at the individual level [43], gene expression studies of several marine species [31], content of Se and Hg in different tissues of alfonsino [41], and growth of Arctic charr, with coefficients of variation between 14 and 69% [44]. The coefficient of variation does not seem to depend on the type of analysis performed or on the level of complexity of the structure analyzed; rather, due to the fractal nature of biological systems, it probably reflects the actual variability of the system [45].

Furthermore, although no studies have been published regarding sea bass, pre- and peri-natal exposure to Hg has effects on selenoprotein activity that may be age-, sex-, and species-dependent. Thus, glutathione peroxidase activity in rats was more than double in hepatic mitochondria from females than from males of the same age [46], and in four species of freshwater fish (*Ambloplites rupestris*, *Lepomis auritus*, *Lepomis gibbosus*, and *Lepomis macrochirus*), the rate of Hg accumulation was found to be significantly faster and the mean Hg levels to be higher in females than in males after the onset of reproduction, although not earlier. On the other hand, previous works had found that males contained higher Hg levels than females in other species [47] and references therein). In mice, exposure to MeHg^+^ during prenatal and lactational periods influenced in a sex- and brain anatomical structure (cerebrum and cerebellum)-specific manner the responses of TrxR, Trx, and GPx [39].

In the present work, the ubiquitousness of Hg (found both in the feed and in the wild hake) not only made it impossible to provide a real control group fed no Hg at all, but also suggests that the broodstock from which these sea bass originated had also been fed Hg-containing feeds. Accordingly, the experimental sea bass used in this work had most likely already been exposed to Hg contamination in ovo, albeit probably to low levels and in conditions of a high Se/Hg molar ratio (given the Se/Hg molar ratios of the feed). The lack of gonad development in our fish did not allow us to determine their sex. Consequently, although both pre-hatch exposure to MeHg^+^ and sex may have influenced the variability of our results, we cannot determine whether they actually did it or to what extent. Further studies should be carried out in the future to clarify these issues.

## Conclusions

Although the treatments used in this work did not produce statistically significant results, both hypotheses were confirmed: (1) all the parameters examined (fish growth and hepatic redox activities, and selenogene expression) were affected by feeding MeHg^+^, and (2) the inclusion of TH in the feed mitigated some of those effects. A third, and most relevant conclusion, is that the effects on redox enzymatic activities and gene transcription were dependent on the Se/Hg molar ratio of the feed but did not follow a linear dose–effect response pattern. Our work strongly supports previous studies [2, 48] evidencing the need to consider together the levels of both Se and Hg in seafood safety, as well as the Se molecular form, when assessing Hg toxicity and endorses the use of Se/Hg molar ratio or some of the proposed parameters (i.e., the HBVSe) by the authorities to establish the safety of food products, rather than continue with the current praxis of referring only to the Hg levels. Two additional important outcomes of this study are (4) the realization that it may be very difficult to carry out experimental works in which Hg is absent, given the ubiquitous nature of the contaminant, and (5) the need to non-invasively monitor fish behavior to identify deviations from the norm that could tip the fish farmer of problems that may end up in poor production or decreased quality, welfare, and wholesomeness of the fish, illustrated in this work by group A3.

## Supplementary Information

Below is the link to the electronic supplementary material.

Supplementary file1 (DOCX 75.1 KB)

## Data Availability

Raw data will be available upon request.
