# Supplementary material for: Antioxidant Activities and Selenogene Transcription in the European Sea Bass (Dicentrarchus labrax) Liver Depend, in a Non-linear Manner, on the Se/Hg Molar Ratio of the Feeds
Source: Biol Trace Elem Res. 2021 Jul 31;200(5):2365–79. doi: 10.1007/s12011-021-02835-7 (PMC9023391; doi:10.1007/s12011-021-02835-7)
Supplement: Supplementary file 1 — Supplementary file1 (DOCX 75.1 KB) [file 12011_2021_2835_MOESM1_ESM.docx]

Antioxidant activities and selenogene transcription in the European sea bass (*Dicentrarchus labra*x) liver depend, in a non-linear manner, on the Se/Hg molar ratio of the feeds.

Marinelle Espino, Harkaitz Eguiraun, Oihane Diaz de Cerio, José Antonio Carrero, Nestor Etxebarria and Iciar Martinez.

***Supplementary Material***

**Supplementary Table 1.** Summary of the BLASTx homologies of the sequences selected from the *D. labrax* genome available at the NCBI (ID). The table includes the location (From, To) of the sequences considered for the homology search. The Txn1 sequence was further analyzed to confirm the family membership.

|  | ID | From | To | Blastx |
| --- | --- | --- | --- | --- |
| *txn1* | FN566754 - Dl_ThNor *Dicentrarchus labrax* cDNA clone Dl_ThNor_40K01, mRNA sequence | 1 | 489 | [Thioredoxin [*Morone saxatilis*]](https://blast.ncbi.nlm.nih.gov/Blast.cgi#alnHdr_XP_035530950) [XP_035530950.1](https://www.ncbi.nlm.nih.gov/protein/XP_035530950.1?report=genbank&log$=prottop&blast_rank=1&RID=6V529XPH013) |
| *gpx1* | DT044993.1 - L579t3 *D. labrax* larval cDNA *D. labrax* cDNA similar to glutathione peroxidase (gpx gene), mRNA sequence | 22 | 570 | [Glutathione peroxidase 1a [*Micropterus salmoides*]](https://blast.ncbi.nlm.nih.gov/Blast.cgi#alnHdr_XP_038553148) XP_038553148.1 |
| *txnrd2* | [GFJW01016941.1](https://www.ncbi.nlm.nih.gov/nuccore/GFJW01016941) - TSA: *D. labrax* 1073169 transcribed RNA sequence | 114 | 502 | \|  \| [*Thioredoxin reductase 2* [*M. saxatilis*]](https://blast.ncbi.nlm.nih.gov/Blast.cgi#alnHdr_XP_035514293) \| \| --- \| --- \| |
| *txnrd3* | GFJX01019781.1 - [TSA: D. labrax 839132 transcribed RNA sequence](https://www.ncbi.nlm.nih.gov/nuccore/GFJX01019781.1/) | 1 | 2345 | Thioredoxin reductase 3 (txnrd3), transcript variant X2, mRNA [[*M. saxatilis*]](https://blast.ncbi.nlm.nih.gov/Blast.cgi#alnHdr_1880367201) |

**Supplementary Table 2.** Sequence homology analysis (BLASTn) of the PCR amplicons.

| Amplicon | BlastN hit | | | | | |
| --- | --- | --- | --- | --- | --- | --- |
|  | Name | NCBI ID | Identity percentage | E-value | Coverage |  |
| *txn1* | Thioredoxin (txn), mRNA [[*M. saxatilis*]](https://blast.ncbi.nlm.nih.gov/Blast.cgi#alnHdr_1880391175) | [XM_035675057.1](https://www.ncbi.nlm.nih.gov/nucleotide/XM_035675057.1?report=genbank&log$=nucltop&blast_rank=1&RID=6T11EDFG016) | 86.17% | 2e-16 | 78% |  |
| *gp 1x* | [Glutathione peroxidase [*D. labrax*]](https://blast.ncbi.nlm.nih.gov/Blast.cgi#alnHdr_CBN80579) | [CBN80579.1](https://www.ncbi.nlm.nih.gov/protein/CBN80579.1?report=genbank&log$=prottop&blast_rank=1&RID=6T0RU97K016) | 96.23% | 1e-27 | 71% |  |
| *txnrd3* | [Thioredoxin reductase 3 [*D. labrax*]](https://blast.ncbi.nlm.nih.gov/Blast.cgi#alnHdr_CBN80599) | [CBN80599.1](https://www.ncbi.nlm.nih.gov/protein/CBN80599.1?report=genbank&log$=prottop&blast_rank=1&RID=6T0TENTA013) | 100.00% | 2e-43 | 98% |  |
| *txnrd2* | Thioredoxin reductase 2 [*M. salmoides*] | [XM_038703974.1](https://www.ncbi.nlm.nih.gov/nucleotide/XM_038703974.1?report=genbank&log$=nucltop&blast_rank=2&RID=6T2H26Z5013) | 93.75% | 2e-08 | 52% |  |

**Supplementary Text Box 1**

**Location of the selected sequences for the design of the primers and how the sequences were sorted as family members for the gene *txn1.* Fasta sequences of the Txn1** gene (from FN566754.1 FN566754 Dl_ThNor *Dicentrarchus labrax* cDNA clone Dl_ThNor_40K01, mRNA sequences). The primers were selected from the sequence below: the yellow sections show non coding domains and the sequences selected to design the primers are indicated in bold and underlined.

GCACTCCCGACTAAACCGTTAGTCGTTCAGTCTCGAGGCACGTTAACTCAACCGTCATCATGGTCCGAGAGGTAGAAAGCCTGGATGACTTCAAGGCCATCCTGAAGGAAGCCGGAGACAAG**CTGGTGGTGGTGGACTTCA**CAGCCACATGGTGTGGCCCCTGTAAACAGATTGGCCCAATATTTGAAGAACAGTCGTTGAAGCCTGAGAACAAGAATGTGATTTTCCTGAAGGTGGACGT**AGATGAGGCTGAGGATGTGAG**TTCATCCTGCAATATAAGTTGCATGCCCACATTCCAGTTTTACAAGAATGGAGAGAAGGTGTTCGAGTTCTCTGGTGCAAACACAAATACGCTGTTGGAAAAACTGGTAGCTTTCAGAACATAAGAACCATAGTAGCCACCGCTGCCCTCTGTACATGCTGTGCTAAGAAATCCCAGTTTCATTTTCTGTACAGTAACTATCAGGTTTCCTGTTGGTGACATACTGT

**Supplementary Table 3**. CLUSTALW 2.1 multiple sequence alignment analysis to verify txn (FN566754) membership of the protein sequence of the gene *txn*, and B) hylLM boostraped of the compared sequences for the indicated species and gene IDs: for txn2 (XP_012681376, *Clupea harengus*; NP445783.1, *Rattus norvegicus*; XP_008413619- *Poecilia reticulate*; NM_205641.1-*Danio rerio*); and for genes belonging to txn1 (NM_053800.3 –*Rattus norvegicus*; NM_001002461.1, *Danio rerio* and AY242060.1, *Melopsittacus undulates*).

| Txn2 | | |
| --- | --- | --- |
| *Danio rerio* | NM_205641.1_33-533 | MAFRLLARRVSRISVKDVRVLVLPSSSSSRSSFSSCFRAAPPLLSRS--I |
| *Clupea harengus* | XP_012681376.1 | MAHRLLVRRIWTVSVRDVRCPRAPSSALYSTSLCSSASPQPLLSPRRSLT |
| *Poecilia reticulate*; | XP_008413619.1 | MAHRLLARRIWTLSVKDVRCLPSSAVTSSSFSTSLHPVSSRASFLAPSRT |
| *Rattus norvegicus* | NP_445783.1 | MAQRLLLRRFLTS-VISRKPPQGVWASLTSTSLQTPPYNAGGLTGTPSPA |
|  |  |  |
| Txn1 | | |
| *D.labrax*_thioredoxin |  | -------------------------------------------------- |
| *Danio rerio* | NM_001002461.1_61-384 | -------------------------------------------------- |
| *Rattus norvegicus* | NM_053800.3 | -------------------------------------------------- |
| *Melopsittacus undulates* | Y242060.1_124-441 | -------------------------------------------------- |
|  |  |  |

| Txn2 | | |
| --- | --- | --- |
| *Danio rerio* | NM_205641.1_33-533 | PRLPYITSRSVSFNVQDHDDFTERVINS-ELPVLIDFHAQWCGPCKILGP |
| *Clupea harengus* | XP_012681376.1 | RALPVTSRRQVSFNVQDQEDFTERVINS-ELPVVIDFHAQWCGPCKILGP |
| *Poecilia reticulate* | XP_008413619.1 | PAVTHAVRRAVSFNVQDNEDFTERVINS-DLPVLVDFHAQWCGPCKILGP |
| *Rattus norvegicus* | NP_445783.1 | RTFHTTRVCSTTFNVQDGPDFQDRVVNS-ETPVVVDFHAQWCGPCKILGP |
| Txn1 | | |
| *D.labrax_*thioredoxin |  | ----------MVREVESLDDFKAILKEAGDKLVVVDFTATWCGPCKQIGP |
| *Danio rerio* | NM_001002461.1_61-384 | ----------MVLEIEDKAAFDNALKNAGDKLVVVDFTATWCGPCQTIGP |
| *Rattus norvegicus* | NM_053800.3 | ----------MVKLIESKEAFQEALAAAGDKLVVVDFSATWCGPCKMIKP |
| *Melopsittacus undulates* | AY242060.1_124-44.1 | ----------MVKSVGCLSEFKAELQSAGENLVVVDFSATWCGPCKMIKP |
|  |  | : * : : : *::** * *****: : * |

| *Txn2* |  |  |
| --- | --- | --- |
| *Danio rerio* | NM_205641.1_33-533 | RLEKAIAKQKG-RVTMAKVDIDEHTDLAIEYGVSAVPTVIAMRGGDVIDQ |
| *Clupea harengus* | XP_012681376.1 | RLEKAIGKQKG-RVTMAKVDIDEHTDLAIEYGVSAVPTVIAMRGGDVIDQ |
| *Poecilia reticulate* | XP_008413619.1 | RLEKAVAKQKG-RVAMAKVDIDDHTDLAIEYGVSAVPTVIAMRGGDVVDR |
| *Rattus norvegicus* | NP_445783.1 | RLEKMVAKQHG-KVVMAKVDIDDHTDLAIEYEVSAVPTVLAIKNGDVVDK |
| Txn1 | | |
| *D.labrax*_thioredoxin |  | IFEEQSLKPENKNVIFLKVDVDEAEDVSSSCNISCMPTFQFYKNGEKVFE |
| *Danio rerio* | NM_001002461.1_61-384 | YFKLLSEKPENKNVVFLKVDVDDAQDVAALCGISCMPTFHFYKNGKKVDE |
| *Rattus norvegicus* | NM_053800.3 | FFHSLCDKYS--NVVFLEVDVDDCQDVAADCEVKCMPTFQFYKKGQKVGE |
| *Melopsittacus undulates* | AY242060.1_124-441 | FFHSLCEKYG--DVVFIEIDVDDAQDVAAHCDVKCMPTFQFYKNGKKVQE |
|  |  | :. * * : ::*:*: *:: :..:**. : *. : . |

| Txn2 | | |
| --- | --- | --- |
| *Danio rerio* | NM_205641.1_33-533 | FVGIKDEDQLDTFVEKLIGQ- |
| *Clupea harengus* | XP_012681376.1 | FVGIKDEDQLDSFVKKLIGQ- |
| *Poecilia reticulate* | XP_008413619.1 | FVGIKDDDQLDSFVCKVIGQ- |
| *Rattus norvegicus* | NP_445783.1 | FVGIKDEDQLEAFLKKLIG-- |
| Txn1 | | |
| *D.labrax_*thioredoxin |  | FSGANTNTLLEKLVAFRT--- |
| *Danio rerio* | NM_001002461.1_61-384 | FSGSNQSKLEEKINSHK---- |
| *Rattus norvegicus* | NM_053800.3 | FSGANKEKLEATITEFASCSE |
| *Melopsittacus undulates* | AY242060.1_124-441 | FSGANKEKLEETIKSLV---- |
|  |  | * * : . : |

**Supplementary Figure 1.** hylLM boostraped analysis of the sequences compared in Supplementary Table 3, for the indicated species and gene IDs: for txn2 (XP_012681376, *Clupea harengus*; NP445783.1, *Rattus norvegicus*; XP_008413619- *Poecilia reticulate*; NM_205641.1-*Danio rerio*); and for genes belonging to txn1 (NM_053800.3 –*Rattus norvegicus*; NM_001002461.1, *Danio rerio* and AY242060.1, *Melopsittacus undulates*).


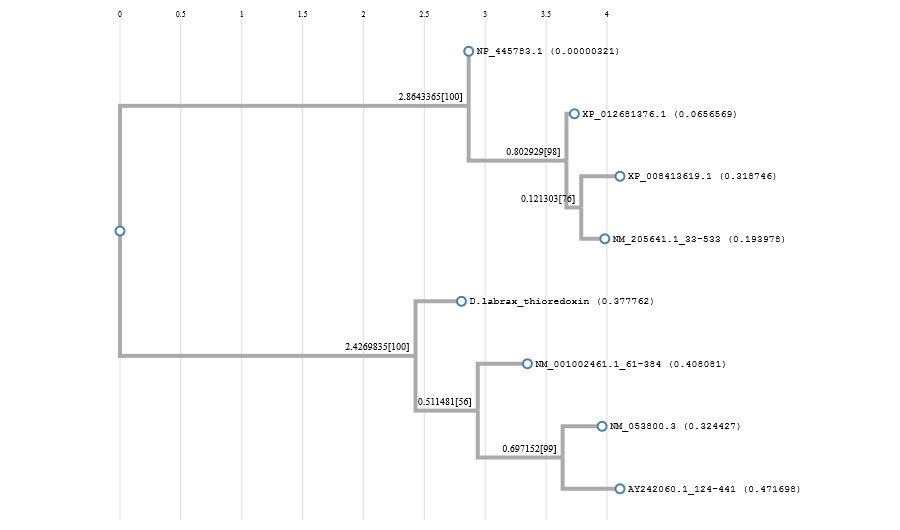


*txn2*

*txn1*
